# Comprehensive evaluation of the relationship between biomarker profiles and neoadjuvant chemotherapy outcomes for breast cancer patients

**DOI:** 10.1186/s13000-024-01451-y

**Published:** 2024-03-20

**Authors:** Mijia Wang, Zhendong Wei, Jixia Kong, Haidong Zhao

**Affiliations:** The Second Hospital of Dalian Medical University, Dalian, 116023 China

**Keywords:** Breast cancer, Neoadjuvant chemotherapy, Biomarkers, pCR, Tumor size change

## Abstract

**Background:**

Accurately predicting the response to neoadjuvant chemotherapy (NAC) in breast cancer patients is crucial for guiding treatment strategies and enhancing clinical outcomes. Current studies have primarily focused on a limited set of biomarkers. More importantly, the results of many studies are in conflict. To address this, we conducted a comprehensive evaluation of the predictive value of a diverse range of clinically available molecular biomarkers in breast cancer, including HER2, ER, PR, TOPO II, EGFR, Ki67, CK5/6, AR, and p53. Additionally, we assessed changes in these biomarkers after NAC administration.

**Methods:**

Our study involved 189 patients with invasive breast cancer who underwent NAC at our institute. We examined biomarker profiles in core-needle biopsies taken before NAC and in surgical specimens obtained after NAC. We examined the association between these biomarkers and NAC outcomes, focusing on two main aspects: the rate of pathological complete response (pCR) and the reduction in tumor size. We used Chi-square and Mann-Whitney U tests to compare biomarker status changes between pCR and non-pCR patients. Linear regression analysis was employed to evaluate the relationship between biomarker status and tumor shrinkage rate. Additionally, we compared the expression status of these biomarkers before and after NAC using Chi-square and Wilcoxon signed-rank tests.

**Results and conclusions:**

Our results demonstrated significant differences in the expression levels of HER2, ER, PR, TOPO II, EGFR, and Ki67 between pCR and non-pCR patients, underscoring their potential as predictive markers for NAC outcomes. Importantly, our results have shed light on the contentious issue surrounding TOPO II in NAC outcome prediction. We have provided evidence that establishes a significantly positive association between TOPO II expression level and the pCR rate. Notably, tumor size was identified as a relevant predictive factor for achieving pCR. Regarding biomarker profiles, only Ki67 levels and TOPO II status exhibited changes following NAC, resolving previous controversies. While the ER and PR status remained unchanged, their expression values exhibited a slight but significant decrease post-NAC. Our results provide clarity and insights into the value and potential of using these biomarkers to predict NAC responses and prognosis in breast cancer patients.

**Supplementary Information:**

The online version contains supplementary material available at 10.1186/s13000-024-01451-y.

## Introduction

Breast cancer is still the leading cause of cancer related death and the most common malignancy among women worldwide, despite recent improvements in terms of prevention, diagnosis and treatment [1, 2]. Currently neoadjuvant chemotherapy (NAC) has been widely used as a standard treatment for high-risk early-stage or locally advanced breast cancer [3]. NAC could reduce tumor stage, improve the chance of breast conservative surgery and eliminate possible micrometastasis [2, 4–6]. Since breast cancer is a heterogeneous disease that presents in various clinical and histological forms, the prognoses and outcomes after NAC can vary greatly. The prediction of response to NAC could facilitate the selection of patients as well as therapeutic strategies. Numerous studies have demonstrated that patients with favorable clinical outcomes, especially those achieving a pathological complete response (pCR), experience significantly improved disease-free survival and overall survival rates [2, 7]. This observation is particularly true in triple-negative and human epidermal growth factor receptor 2 (HER2) positive, hormone-receptor-negative breast cancers [8]. However, despite being a primary goal of NAC, achieving pCR remains limited to a minority of patients [9]. Due to the lack of large-scale comprehensive analysis, it is still unclear which patients are more likely to achieve pCR and better outcomes after NAC.

Over the past decades, the exploration of molecular biomarkers has significantly advanced our understanding of the heterogeneous nature of breast cancer. Notably, three predominant biomarkers—estrogen receptor (ER), progesterone receptor (PR), and HER2—have become essential in clinical practice for identifying intrinsic breast cancer subtypes and guiding treatment decisions. Investigations into the correlation between the prominent biomarkers and pCR rates after NAC have predominantly focused on these three markers. Additionally, conflicting results have been observed in previous studies on this issue [2]. Therefore, there is a need for further analysis to reconcile these discrepancies and broaden our investigation to include additional biomarkers in predicting pCR following NAC. Furthermore, although tumor size reduction is widely recognized as a key indicator of NAC response, there is a notable scarcity of studies that delve into the link between biomarkers and changes in tumor size after NAC [10]. Finally, it would be interesting to explore whether the reassessment of biomarker status is essential post-NAC for the purpose of adjusting therapies. The variable and occasionally conflicting outcomes from empirical studies on how chemotherapy impacts biomarker expression underscore the need for additional investigation to guide the clinical application of these molecular biomarkers [11–20].

To address these challenges, this retrospective study conducts a comprehensive analysis of the correlation between molecular biomarkers and NAC outcomes in breast cancer patients. Specifically, we explore the predictive potential of these biomarkers for both the pathological pCR rate and tumor shrinkage. Our study extends beyond previous research by examining nine clinically available biomarkers, including two hormone receptors (ER and PR), HER2, topoisomerase II (TOPO II), epidermal growth factor receptor (EGFR), the cellular proliferation index (Ki67), cytokeratin 5/6 (CK5/6), androgen receptor (AR), and tumor suppressor protein 53 (p53). Furthermore, we investigate how the status of these biomarkers changes after NAC administration. Our study provides a unique and robust perspective for understanding the predictive and prognostic value of these biomarkers in the context of neoadjuvant chemotherapy for breast cancer patients.

## Methods

### Patient data collection

This retrospective study included 189 breast cancer patients, who underwent NAC at the Second Hospital of Dalian Medical University between 2016 and 2022. The study protocol conformed to the ethical guidelines of the 1975 Declaration of Helsinki was approved by the Medical Ethics Committee of Dalian Medical University Cancer Institute and Hospital. Patients were enrolled if they met the following eligibility criteria: (1) underwent at least four cycles of NAC; (2) had surgery after NAC; (3) completed ultrasound examination in all treatment cycles; (4) had no distant metastasis; (5) had no occult breast cancer; (6) had no cN3; (7) had no inflammatory breast cancer. These patients were treated with one of the following chemotherapies. (1) cytotoxic, which includes AC (doxorubicin and cyclophosphamide), taxanes and/or platinums; (2) cytotoxic + trastuzumab or cytotoxic + trastuzumab + patozumab. The dosage was calculated based on the patients’ body surface area or body weight: doxorubicin 75-100mg/m^2^; cyclophosphamide 600mg/m^2^; taxanes 175mg/m^2^; platinums AUC = 6; trastuzumab 8 mg/kg for the initial treatment followed by subsequent treatments at a dosage of 6 mg/kg. Regarding patozumab, an initial dose of 840 mg was administered, followed by subsequent doses of 420 mg.

We collected clinical information including age, before or after NAC clinical tumor stage and pathological information. The pathological information included status or expression levels for biomarkers including ER, PR, HER2, TOPO II, EGFR, Ki-67, CK5/6, AR and p53. TNM tumor staging was performed according to the 8th edition of the American Joint Committee on Cancer staging system. pCR was defined as no invasive disease in the breast and no metastatic in axillary lymph nodes. The patients achieving pCR after NAC could not undergo immunohistochemistry to collect the pathological information.

### Immunohistochemistry

We collected core needle biopsies for all the patients before NAC (mentioned as biopsies before NAC) and prepared postoperative breast specimens (mentioned as surgical specimens after NAC) for patients who did not achieve pCR after NAC, following College of American Pathologists (CAP) protocols. Tumor tissues with a component of over 95% tumor cells were analyzed for biomarkers using immunohistochemistry (IHC). IHC staining was performed on both needle biopsies and surgical specimens using the EnVision two-step method following the manufacturer’s instructions. All the antibodies were purchased from Beijing Zhongshan Golden Bridge Biotechnology. PBS was used as a negative control for the primary antibody staining. Hormone receptors (ER and PR), AR, p53 and Ki67 expression values were calculated as the percentage of positive nuclear staining in the IHC slide evaluated. Status of hormone receptors was considered positive + when they exceeded 1% of nuclear staining in tumor cells [21]. HER2 level was determined following the recommendations of the American Society of Clinical Oncology (ASCO)/CAP guideline [22]: level 3 for complete and intense circumferential membrane within > 10% of tumor cells; ambiguous level 2 for incomplete and/or weak/moderate circumferential membrane staining within > 10% of tumor cells, or complete membrane staining but within ≤10% of tumor cells; level 1 for incomplete faint membrane staining within > 10% of tumor cells; and level 0 for absence of staining. HER2 status (classified as either negative or positive) was further delineated based on both the HER2 level and FISH testing using the following criteria: HER2-positive was defined as level 3 or level 2 with positive FISH testing, while HER2-negative was defined as level 0, level 1, or level 2 with negative FISH testing [23]. Ki67 expression ≥20% was considered high level as suggested [24]. TOPO II level was determined as follows: level 0 for absence of staining, level 1 for nuclear staining within < 25% of tumor cells; level 2 for nuclear staining within ≥25% and < 50% of tumor cells; level 3 for nuclear staining within ≥ 50% and < 75% of tumor cells; level 4 for nuclear staining within ≥75% of tumor cells. EGFR level was determined based on the percentage of cytoplasmic/membrane staining of tumor cells: - if no staining observed; + for staining within < 25% of tumor cells; ++ for staining ≥25% and < 50%; +++ if staining ≥50%. CK5/6 was considered positive + if > 10% of tumor cells showed cytoplasmic staining, and negative - if no staining or ≤10% of tumor cells were stained [25].

### Statistical analyses

We compared the expression status of biomarkers in core needle biopsies between pCR and non-pCR patients using (1) Chi-square test for categorical variables; and (2) Mann-Whitney U test for continuous variables since the sample distributions were not normally distributed. We evaluated the relationship between tumor size change and biomarker status before NAC using linear regression analysis. The tumor size change was represented as the ratio of tumor size before to tumor size after NAC. This ratio was used as a response variable, while the biomarkers were used as predictor variables for regression analysis. We performed linear regression analysis for dummy variable for categorical biomarkers such as ER, PR, HER2, TOPO II, EGFR and CK5/6.

We analyzed changes in biomarker expression status or tumor size in paired samples from both needle biopsies and surgical specimens using (1) Chi-square test for categorical variables; and (2) Wilcoxon signed-rank test for continuous variables since the sample distributions were not normally distributed.

We conducted statistical analyses using python3 scipy package (version 1.7.3): chi2_contingency function for Chi-square test, shapiro function to test the distribution of continuous variable, mannwhitneyu function for Mann-Whitney U test and wilcoxon function for Wilcoxon signed-rank test. We performed the linear regression analysis using python3 statsmodels package (version 0.13.2) api function. Differences or linear relationship were considered statistically significant when *p*-value < 0.05.

## Results

### Characterization of patients and study summary

We conducted a retrospective review of 189 patients diagnosed with breast cancer between 2016 and 2022 at our institution. The age of patients in our study ranged from 24 to 78 years, with a median age of 51 years. All these patients received NAC. The NAC consisted mostly of cytotoxic chemotherapy (Table 1). We measured the tumor size and examined lymph node metastasis, and therefore staged the tumors following the TNM classification as summarized in Table 1. In our study, there were 26 out of 189 patients having no residual invasive carcinoma or ductal carcinoma in situ in breast or lymph nodes. Therefore, 14% of patients achieved pCR after NAC administration.

We examined the status of 9 clinically available biomarkers used for monitoring or decision making from both needle biopsies (hereafter referred as biopsies before NAC) and the resection specimens after receiving NAC (hereafter referred as after/post NAC). Our analysis included ER, PR, HER2, TOPO II, EGFR, Ki-67, CK5/6, AR and p53. We will discuss the results later to evaluate the predictive value of these biomarkers.

**Table 1:** Clinical characteristics of the 189 patients included in the study

Tumor stage	Before NAC				After NAC			
	T0	T1	T2	T3	T0	T1	T2	T3
	0	19	131	39	30	61	69	29
Histopathology	Ductal		Lobular				Others	
	179		3				7	
Involvement of lymph nodes	Before NAC				After NAC			
	Yes		No		Yes		No	
	129		60		112		77	
Type of NAC	Cytotoxic		Cytotoxic + trastuzumab or Cytotoxic + trastuzumab + patozumab					
	163		26					

Biomarkers of core-needle biopsies and surgical specimens are referred as before NAC and after NAC, respectively

### Tumor size reduction after receiving NAC

As shown in Table 1, the number of patients with T0, T1, T2 and T3 stage breast cancers before NAC were 0, 19, 131 and 39, respectively. Our results indicated that NAC significantly reduced the size of tumor lumps: most carcinomas were reduced in size (85%, 160/189), and the mean tumor size was 3.78 cm before NAC vs. 2.29 cm after NAC (*p* < 0.01, Wilcoxon signed-rank test) (Supplementary Fig. 1 and Table 2). We observed 101 cases with downstaged tumors after NAC. In particular, 30 cases were with T0 stage after treatment, including 26 patients having no residual tumor, achieving pCR, and 4 patients having invisible tumor but observed lymph node metastasis.

**Table 2:** Comparison of the before NAC biomarker status between pCR and non-pCR patients

	pCR				non-pCR				Test	*n*	*p*-value
HER2	0	1	2	3	0	1	2	3	Chi-square	189	<0.001*
	3	2	2	19	13	43	64	43			
ER	-		+		-		+		Chi-square	189	<0.001*
	23		3		41		122				
PR	-		+		-		+		Chi-square	189	<0.001*
	25		1		49		114				
TOPO II	0	1	2	3	0	1	2	3	Chi-square for Ordinal Data	118	0.03*
	0	2	5	2	1	58	43	7			
EGFR	-	+	++	+++	-	+	++	+++	Chi-square	120	<0.001*
	1	1	6	1	52	39	16	4			
Ki67	Mean = 52.81				Mean = 36.40				Mann-Whitney U	189	<0.001*
CK5/6	-		+		-		+		Chi-square	116	0.23
	4		3		84		25				
AR	Mean = 42.7				Mean = 35.80				Mann-Whitney U	134	0.78
p53	Mean = 43.88				Mean = 26.86				Mann-Whitney U	119	0.07
Tumor size	Mean = 2.75 cm				Mean = 3.94 cm				Mann-Whitney U	189	<0.001*

*n* is the number of pCR and non-pCR patients having the according information and used for analysis

For categorical biomarkers HER2, ER, PR, TOPO II, EGFR, CK5/6, the number of patients in each category was listed. The HER2 and TOPO II have 4 grading levels: level 0 to level 4. The EGFP have 4 grading levels labeled with -, +, ++ and +++

For biomarkers with continuous values such as Ki67, the mean value was shown in the table

*differences are considered statistically significant at a *p*-value of less than 0.05

### Comparison of biomarker status between pCR and non-pCR patients

Achieving pCR to NAC has been shown to be associated with improved patient outcomes, however it occurs only in a minority of patients. To determine whether these molecular biomarkers can provide prognostic information or predict response to NAC, we compared the expression status/levels of 9 biomarkers in needle biopsies between patients achieving pCR (pCR subgroup) and those not (non-pCR subgroup). First, we performed Chi-square test to compare the biomarkers having categorical levels (Table 2). The analysis revealed that the status of HER2, EGRF and TOPO II, which were classified into 4 levels, were all significantly different between the pCR and non-pCR patients. To be specific, the pCR rate was 19% (3/16), 4% (2/45), 3% (2/66) and 31% (19/62) for patients with HER2 IHC expression Level 0, 1, 2 and 3, respectively (*p* < 0.001). Patients with EGRF ++ (27% pCR) and +++ (20% pCR) had better outcomes than those with EGRF − (2% pCR) and + (3% pCR). Remarkably, the TOPO II status was significantly different with a Chi-square test for ordinal data, indicating that high TOPO II level was associated with better pCR rates (*p* < 0.05). Indeed, there were 0% (0/1), 3% (2/60), 10% (5/10) and 22% (2/9) patients experiencing pCR post NAC, corresponding to TOPO II levels from 0 to 3. In addition, we observed that the ER and PR status, which were classified as either negative - or positive +, were also significantly different (*p* < 0.001) between the two patient subgroups. ER negative patients had a significantly higher pCR rate: 36% (23/64) ER negative patients achieving pCR while only 2% (3/125) for ER positive patients. Like ER, PR negative patients also had a better chance for reaching a pCR (34%, 25/74) than the positive ones (1%, 1/115). In contrast, we did not observe any statistically significant difference in CK5/6 status between the pCR and non-pCR patients (*p* = 0.23).

Next, we examined the biomarkers with continuous values using Mann-Whitney U Test (Table 2). We found that the Ki67 expression levels were significantly higher in pCR patients (mean value 53 in pCR vs. 36 in non-pCR, Mann-Whitney U Test, *p* < 0.001). While no differences were observed for the other two continuous biomarkers AR (*p* = 0.78, Mann-Whitney U Test) and p53 (*p* = 0.07, Mann-Whitney U Test) between the two patient subgroups.

Furthermore, given the significant role of tumor stage in chemotherapy response [2], we investigated whether tumor size could serve as a predictor for NAC response. We compared the T stage of tumors in pCR and non-pCR patients before NAC, treating it as a categorical variable. Even though not statistically significant (*p* = 0.15 Chi-square test or *p* = 0.06 Chi-square test for ordinal data), we still observed a trend for higher pCR rate in patients with lower tumor grading. pCR rate was 26%, 14% and 8% in patients having T1, T2 and T3 tumor stage, respectively. This trend is consistent with the observation that pCR patients started with a significantly smaller tumor size before receiving NAC (mean tumor size 2.75 cm for pCR patients vs. 3.94 cm for non-pCR patients, *p* < 0.001 Mann-Whitney U Test, Table 2).

### Relationship between the tumor size change and biomarker status

We further investigated the prognostic potential of these molecular biomarkers for tumor shrinkage in the patients receiving NAC. We estimated the relationship between tumor size reduction (defined as the fold change between before NAC tumor size and after NAC size) and the pre-NAC status/levels of these biomarkers using linear regression analysis for dummy variables (categorical data) or continuous variables (continuous data) (Table 3). The analysis revealed significant correlations between the tumor size change and the expression status of ER, PR and Ki67 (*p* < 0.05 for all three) (Supplementary Fig. 2), indicating that these three biomarkers are promising predictive indicators for tumor shrinkage after NAC administration. Specifically, ER negative patients had a greater chance of obtaining breast tumor shrinkage. We observed tumor downstaged in 67% (43/64) or 46% (58/125) of the ER negative or positive patients, respectively. Like ER, PR negative patients had a higher downstaged rate than the positive ones (70%, 51/74 vs. 43%, 50/115). For Ki67, there was a negative correlation between the size change and its expression value (coef=-0.0044, Table 3), suggesting that a higher Ki67 value was a predictive factor for better response to NAC in terms of tumor size reduction. Indeed, the subgroup with a high Ki67 value ( > = 20%) had 58% of tumor downstaged while the low Ki67 subgroup only had 27%. Whereas for biomarkers HER2, EGFR and TOPO II, although they could be predictive indicators for pCR, they were not associated with the tumor size change (Table 3 and Supplementary Fig. 2). For the other three biomarkers CK5/6, AR and p53, there was no significant correlation observed either (Table 3 and Supplementary Fig. 2).

**Table 3:** Linear regression analysis of the relationship between the tumor size change and biomarkers

	Test	*n*	Coefficient	*p*-value
HER2	Linear regression for dummy variable	189	Not significant	> 0.2
ER	Linear regression for dummy variable	189	0.3184	< 0.001*
PR	Linear regression for dummy variable	189	0.2821	< 0.001*
TOPO II	Linear regression for dummy variable	118	Not significant	> 0.2
EGFR	Linear regression for dummy variable	120	Not significant	> 0.05
Ki67	Linear regression	189	-0.0044	0.003*
CK5/6	Linear regression for dummy variable	116	Not significant	> 0.05
AR	Linear regression	134	Not significant	> 0.5
P53	Linear regression	119	Not significant	> 0.2

*p*-value is the *P* >|t| value associated with the model coefficient(s)

*n* is the number of patients having the according information therefore used for regression analysis

*differences are considered statistically significant at a *p*-value of less than 0.05

### Comparison of biomarkers status change after NAC

Finally, we evaluated whether there were changes in the status of the biomarkers after NAC administration. For this analysis, we chose the patients having biomarker information collected in both the initial biopsy specimens and the resection specimens. We compared the expression status and levels of these molecular biomarkers in paired samples using either Chi-square test or Wilcoxon signed-rank test (Table 4). We found that the Ki67 expression level was significantly decreased after receiving NAC: mean value 35 pre vs. 27 post (*p* < 0.001, Wilcoxon signed-rank test, Table 4, Supplementary Fig. 3). Interestingly, we did not observe any changes in ER nor PR status in paired samples. There were 32 (22% before) vs. 36 (25% after) cases with ER negative (Chi-square test, *p* = 0.58), and 41 (28% before) vs. 46 (32% after) cases with PR negative (Chi-square test, *p* = 0.52), respectively (Table 4). However, the expression level of ER and PR was slightly but significantly lower in the after NAC samples: for ER 52 vs. 49 (*p* = 0.039, Wilcoxon signed-rank test) and for PR mean value 31 vs. 26 (*p* = 0.013, Wilcoxon signed-rank test) (Table 4). In addition, the status of TOPO II was also significantly different (Chi-square test, *p* = 0.041, Table 4). There were 38 out of 84 cases analyzed showing changes in TOPO II status. Accordingly, we observed a trend for downgrade in the TOPO II status in patients after receiving NAC: the percentage of patients with TOPO II level I and level II was 51% and 41% before NAC while 63% and 21% after NAC (Table 4). As for HER2, although 42 out of 143 cases showed changes in status, the difference was not significant (*p* = 0.07, Chi-square test) and the status presented a similar distribution: the percentage with 0, 1, 2 and 3 were 7%, 26%, 43% and 24% before and 2%, 36%, 40% and 22% afterwards. The similar observation went for EGFR (*p* = 0.73, Chi-square test), CK5/6 (*p* = 0.22, Chi-square test), AR (*p* = 0.22, Wilcoxon signed-rank test) and p53 (*p* = 0.93, Wilcoxon signed-rank test) (Table 4).

**Table 4:** Comparison of biomarker profiles before and after NAC

	Before NAC							After NAC							Test	*n*	*p*-value
HER2	0	1	2			3		0	1			2		3	Chi-square	143	0.072
	11	37	61			34		3	51			57		32			
ER	Mean = 52.42							Mean = 49.36							Wilcoxon	146	0.039*
ER	-		+					-				+			Chi-square	146	0.58
	32		114					36				110					
PR	Mean = 31.45							Mean = 22.28							Wilcoxon	146	0.013*
PR	-			+				-		+					Chi-square	146	0.52
	41			105				46		100							
TOPO II	0	1		2	3		4	0	1	2	3		4		Chi-square	84	0.041*
	1	43		35	5		0	4	53	18	8		1				
EGFR	-	+		++	+++			-	+	++	+++				Chi-square	88	0.73
	45	31		9	3			47	29	11	1						
Ki67	Mean = 35.28							Mean = 27.18							Wilcoxon	145	<0.001*
CK5/6	-			+				-		+					Chi-square	86	0.22
	68			18				61		25							
AR	Mean = 33.30							Mean = 29.67							Wilcoxon	99	0.22
p53	Mean = 25.32							Mean = 25.68							Wilcoxon	87	0.93

*n* is the number of patients having paired information (both before and after NAC) therefore used for the according analysis

*differences are considered statistically significant at a *p*-value of less than 0.05

In clinical practice, the assessment of HER2 status, which involves differentiation based on both HER2 level and FISH testing (See Method for details), plays a pivotal role in breast cancer diagnosis and treatment planning. HER2 positive breast cancers exhibit more aggressive behavior and a higher recurrence risk, necessitating targeted therapies like trastuzumab for improved outcomes. Conversely, HER2 negative cases may require different treatment approaches. Recognizing the treatment implications associated with HER2 status, our study delved into specific biomarker status changes in HER2 positive and HER2 negative subtypes. By incorporating FISH testing, we determined the HER2 status of 102 patients as negative and 87 patients as positive before receiving NAC. Subsequent analysis revealed significant differences in ER (Mann-Whitney U Test, *p* < 0.001), PR (Mann-Whitney U Test, *p* < 0.001), and Ki67 (Mann-Whitney U Test, *p* < 0.001) levels between the HER2 positive and negative patients (mean value shown in Supplementary Tables 1 and 2), while other biomarkers showed no subtype-specific variations. Importantly, the alterations in the status of biomarkers after NAC showed differences following HER2 subtyping. As noted previously without distinguishing HER2 status, both HER2 positive and HER2 negative patients exhibited significant decreases in PR and Ki67 expression levels post-NAC; in contrast, ER expression remained unchanged (Supplementary Tables 1 and Supplementary Table 2). Furthermore, AR expression demonstrated significant differences after treatment, specifically in HER2 positive patients, while TOPO II status exhibited significant changes exclusively in HER2 negative patients (Supplementary Tables 1 and 2).

## Discussion

Molecular biomarkers are widely used to help guide breast cancer treatments and prognosis. In this work, we described a comprehensive evaluation of the predictive and prognostic value of 9 clinically available molecular biomarkers for breast cancer. We investigated the predictive potential of these biomarkers for NAC outcomes in two aspects: achieving pCR and shrinking tumor, both of which have been shown to be highly associated with disease-free survival and overall survival rates [10, 26–28]. We found that the expression status or levels of HER2, EGFR, TOPO II, ER, PR and Ki67 were significantly different in pCR patients in comparison to the non-pCR patients. Our findings suggested that negative ER or negative PR or higher Ki67 expression was associated with increasing rates of pCR, which is consistent with the previous results [29–32]. For HER2 status, we adopted an advanced 4 level grading system. We observed that patients with HER2 level 3 had the highest pCR rate, which is similar to the previous report [8, 33]. In addition, patients with HER2 level 0 also had a higher PCR rate than patients with HER2 level 1 or level 2. Numerous studies have shown that more aggressive subtypes including triple negative and high-grade HER2-positive tumors were associated with pCR and better long-term outcomes [34]. Therefore, our observation confirmed this conclusion to some extent. Regarding TOPO II, previous studies have yielded conflicting conclusions. Some studies suggest TOPO II is not predictive for pCR [35, 36]. In contrast, other studies have shown that TOPO II may serve as a predictive biomarker for pCR. Song et al. has reported that negative TOPO II was predictive for pCR [37] while several other analyses have found that response to anthracyclines seems to correlate positively with TOPO II levels [38–41]. With applying a proper statistical model (Chi-square test for Ordinal data), we provided evidence showing a significantly positive association with the pCR rate and TOPO II expression level, indicating it may be a promising prognostic marker as the hormone receptors. In addition, we also confirmed the previous finding that tumor size seemed to be a predictor of complete pathologic response in patients with invasive breast cancer [10].

While tumor shrinkage is widely acknowledged as a key indicator of tumor response to NAC, there is limited evidence regarding the association between biomarkers and tumor size reduction. Our analysis provides a new perspective for understanding the predictive value of these biomarkers on tumor response. We found that only ER, PR and Ki67 these three biomarkers were associated with tumor size reduction, while HER2, EGFR and TOPO II has no significant association. This difference in the predictive potentials of biomarkers on pCR and tumor response is not surprising [10, 42]. Our findings indicate that while tumor size change is a relevant predictive factor for NAC response, it may not be sufficient to predict whether patients will achieve pCR. As for the other biomarkers, CK5/6, AR and p53, we did not observe any significant predictive value for either pCR or tumor response.

It has been reported that the status of some biomarkers may change in surgical specimens after NAC, but the conclusions were controversial. Therefore, we further examined changes in this biomarker status or these expression levels in paired biopsy and surgical samples in breast cancer cases treated with NAC. It has been well described that NAC treatment affects Ki67 expression [16, 43]. Our result is in good agreement with these observations: Ki67 expression was significantly lower after NAC treatment. Regarding ER and PR, the previous findings were mixed, with support found for both variation and no variation after NAC treatment [14, 15, 43–46]. Our result suggested that although the status of both ER and PR were not changed, their expression levels were slightly but significantly altered after NAC treatment. Remarkably, upon more detailed subtyping based on HER2 status, we observed a consistent lack of change in ER expression levels in both HER2 positive and HER2negative patients. This suggests that the expression of estrogen receptor may be influenced by other biomarkers in the context of breast cancer. These findings help explain the inconsistencies reported in previous studies. In addition, HER2 status alternation has been reported to be less common than for Ki67 index and hormone receptors [47]. As in our case, although we observed no significant difference in HER2 status, its p-value was close to the chosen alpha value 0.05, suggesting the conclusion may change with a larger sample size. Furthermore, we noted a significant alteration in TOPO II status after treatment. Notably, AR expression levels exhibited a decrease specifically in HER2 positive patients.

The fact that HER2 subtyping could affect the ER and AR status changes suggests a potential interaction between these biomarkers. Conducting a comprehensive multiple factor analysis would enhance our understanding of how these biomarkers can guide precision medicine treatments. The absence of this multiple factor analysis due to the small clinical sample size is a notable limitation of our study. In particular, the limited number of pCR and triple-negative patients hampers the statistical power for other subgroup analyses. For instance, while CK5/6 did not exhibit a significant value as a standalone factor, previous reports indicate its significance as an adverse prognostic marker in triple-negative breast cancer patients [48]. Therefore, further research, including meta-analysis with a larger sample size, is needed for a more comprehensive evaluation and prediction of NAC outcomes based on biomarkers.

## Conclusion

Our analysis provides a unique perspective on the predictive value of nine clinically available biomarkers for breast cancer in the context of NAC response. The results underscore the predictive potential of two hormone receptors, ER and PR, as well as the cellular proliferation index, Ki67. Patients with negative ER or PR status or higher Ki67 expression exhibit a heightened probability of achieving pathological complete response (pCR) and experiencing tumor reduction following NAC. Conversely, HER2, TOPO II, and EGFR were significantly associated with pCR rates but did not demonstrate correlations with changes in tumor size. In contrast, the biomarkers CK5/6, AR, and p53 did not exhibit predictive potential for NAC outcomes in our study. Moreover, our findings emphasize the relevance of tumor size as a predictive factor for attaining pCR. In examining biomarker profiles, we observed status changes in TOPO II and variations in the levels of Ki67, ER, and PR following NAC administration.

This comprehensive analysis contributes valuable insights to the application of biomarkers within the realm of precision medicine. In summary, our study enhances the understanding of how biomarkers can inform NAC outcomes in breast cancer patients, further guiding the path towards personalized and more effective therapeutic strategies.

### Supplementary Information

**Additional file 1: Supplementary Figure 1.** Tumor size (in cm) before and after NAC administration.

**Additional file 2: Supplementary Figure 2.** Correlation between tumor size change and categorical biomarker status.

**Additional file 3: Supplementary Figure 3.** Paired Wilcoxon signed-rank analysis of expression change of continuous biomarker after NAC.

**Additional file 4: Supplementary Table 1.** Comparison of biomarker profiles before and after NAC for HER2 negative patients.

**Additional file 5: Supplementary Table 2.** Comparison of biomarker profiles before and after NAC for HER2 positive patients.

## Data Availability

No datasets were generated or analysed during the current study.

## Abbreviations

pCR: Pathological complete response
NAC: Neoadjuvant chemotherapy
IHC: Immunohistochemistry
ER: Estrogen receptor
PR: Progesterone receptor
HER2: Human epidermal growth factor receptor 2
TOPO II: Topoisomerase II alpha, EGFR:epidermal growth factor receptor, Ki67:the cellular proliferation index
CK5/6: Cytokeratin 5/6, AR:androgen receptor
p53: Tumor suppressor protein 53
