# Supplementary figures and images for: Comprehensive evaluation of the relationship between biomarker profiles and neoadjuvant chemotherapy outcomes for breast cancer patients
Source: Diagn Pathol. 2024 Mar 20;19:53. doi: 10.1186/s13000-024-01451-y (PMC10953119; doi:10.1186/s13000-024-01451-y)

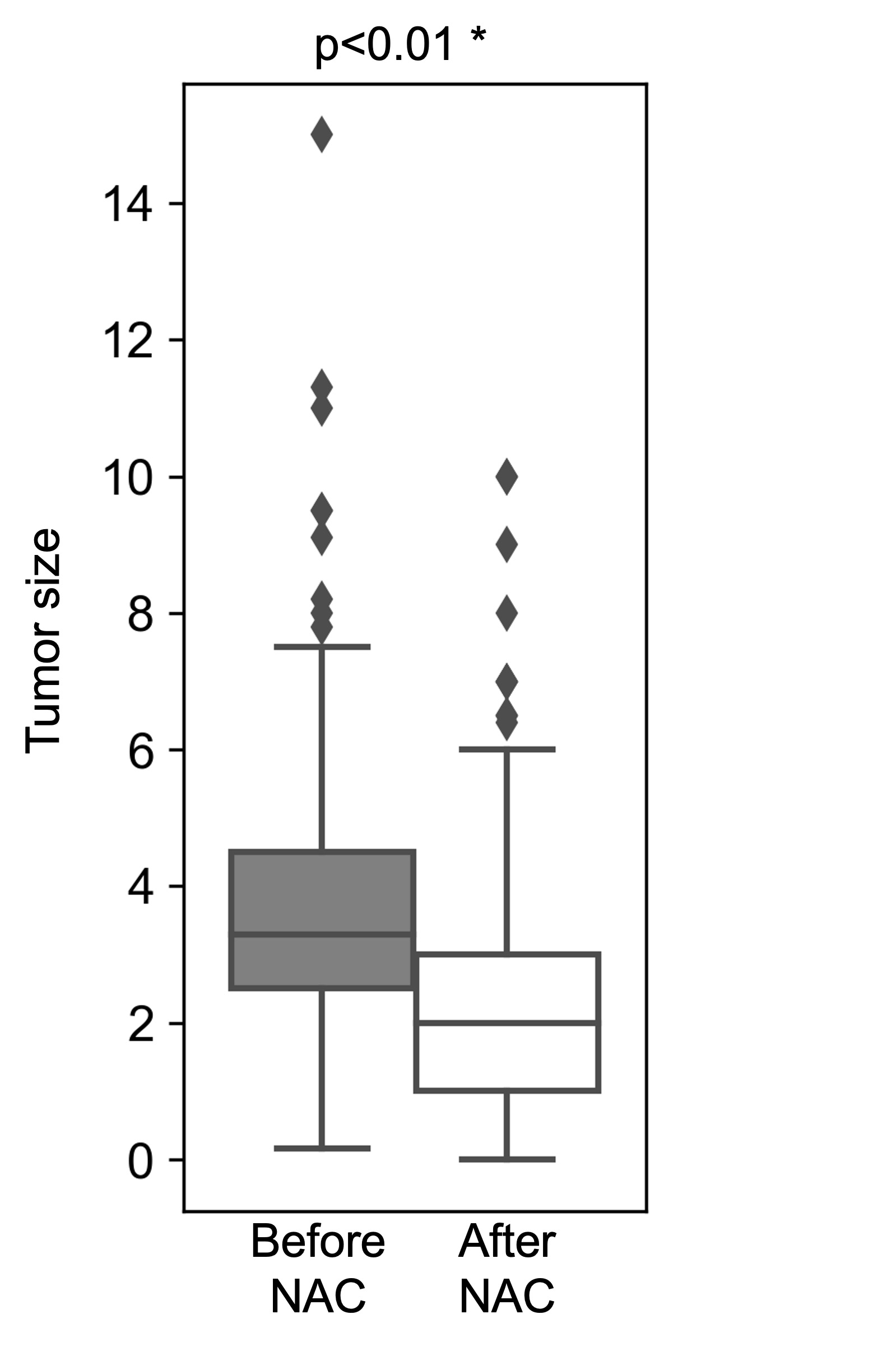

Supplement: Supplementary file 1 — Additional file 1: Supplementary Figure 1. Tumor size (in cm) before and after NAC administration. [file 13000_2024_1451_MOESM1_ESM.jpg]

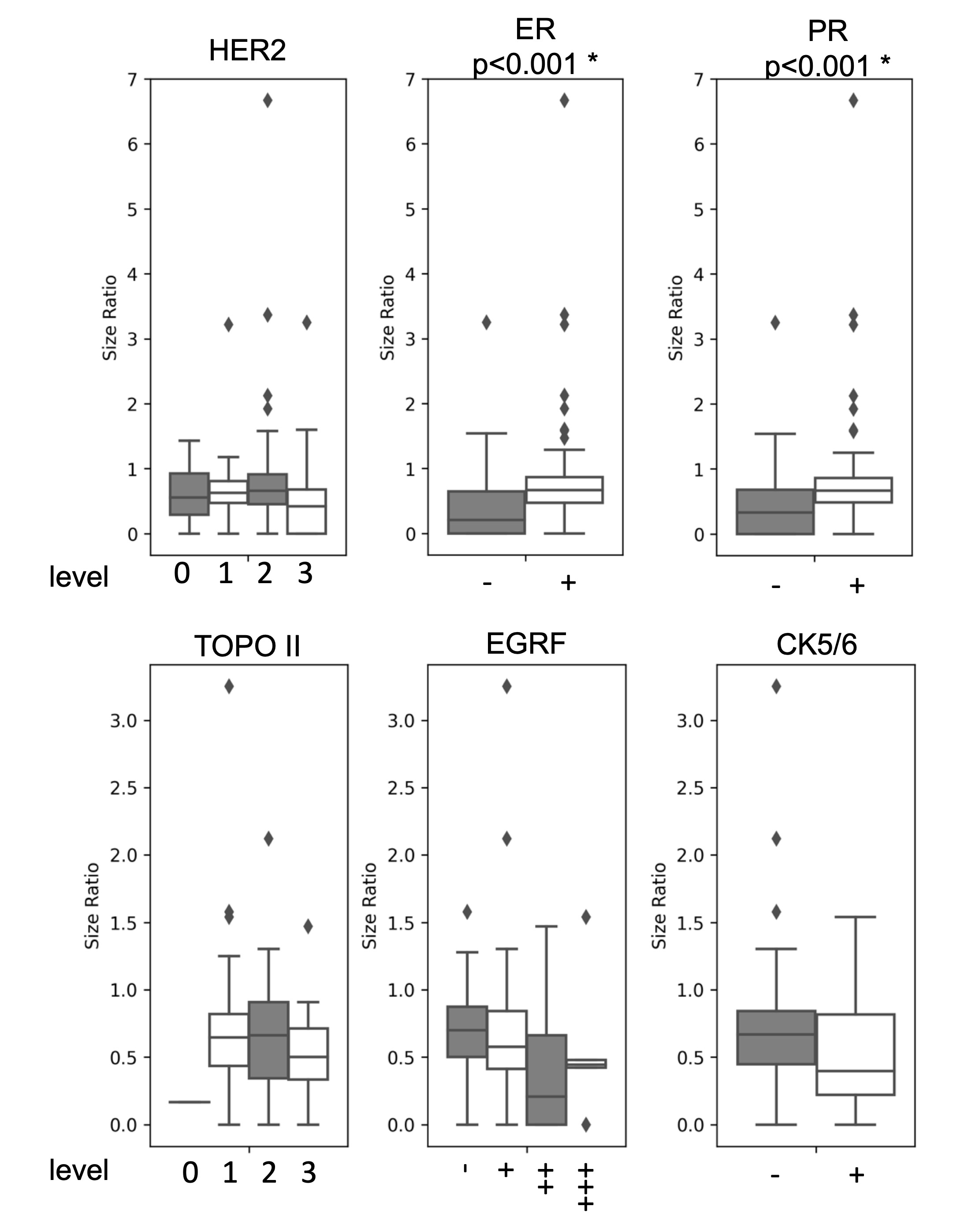

Supplement: Supplementary file 2 — Additional file 2: Supplementary Figure 2. Correlation between tumor size change and categorical biomarker status. [file 13000_2024_1451_MOESM2_ESM.jpg]

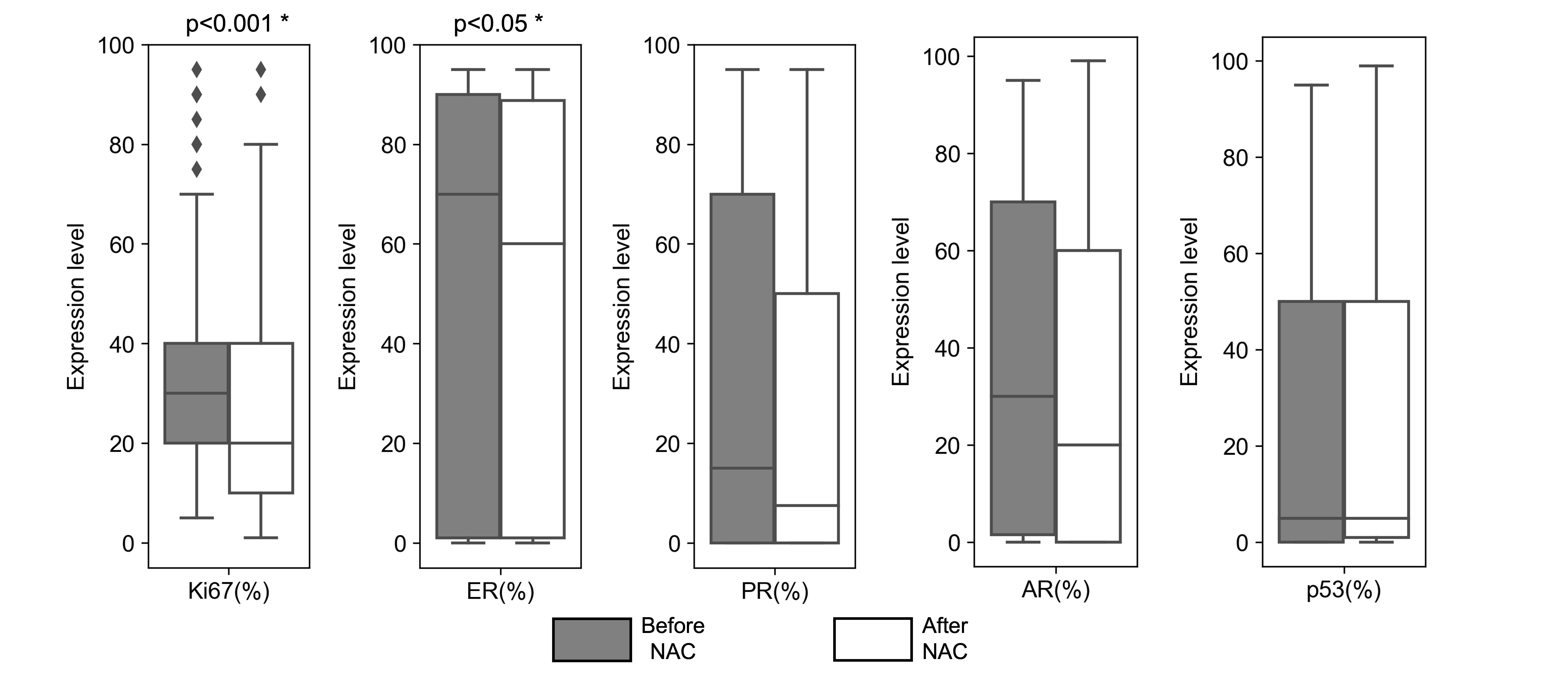

Supplement: Supplementary file 3 — Additional file 3: Supplementary Figure 3. Paired Wilcoxon signed-rank analysis of expression change of continuous biomarker after NAC. [file 13000_2024_1451_MOESM3_ESM.jpg]
