# Supplementary material for: Comprehensive evaluation of the relationship between biomarker profiles and neoadjuvant chemotherapy outcomes for breast cancer patients
Source: Diagn Pathol. 2024 Mar 20;19:53. doi: 10.1186/s13000-024-01451-y (PMC10953119; doi:10.1186/s13000-024-01451-y)
Supplement: Supplementary file 4 — Additional file 4: Supplementary Table 1. Comparison of biomarker profiles before and after NAC for HER2 negative patients. [file 13000_2024_1451_MOESM4_ESM.docx]

Supplementary Table 1: Comparison of biomarker profiles before and after NAC for HER2 negative patients

|  | Before NAC | | | | | | | | After NAC | | | | | | | | Test | n | p-value |
| --- | --- | --- | --- | --- | --- | --- | --- | --- | --- | --- | --- | --- | --- | --- | --- | --- | --- | --- | --- |
| ER | Mean = 64.16 | | | | | | | | Mean = 60.67 | | | | | | | | Wilcoxon | 88 | 0.14 |
| ER | - | | | | + | | | | - | | | | + | | | | Chi-square | 88 | 0.83 |
|  | 12 | | | | 76 | | | | 13 | | | | 75 | | | |  |  |  |
| PR | Mean = 39.70 | | | | | | | | Mean = 33.00 | | | | | | | | Wilcoxon | 88 | 0.026 * |
| PR | - | | | | + | | | | - | | | | + | | | | Chi-square | 88 | 0.70 |
|  | 16 | | | | 72 | | | | 18 | | | | 70 | | | |  |  |  |
| TOPO II | 0 | 1 | | 2 | | 3 | | 4 | 0 | 1 | | 2 | | 3 | | 4 | Chi-square | 54 | 0.013 * |
|  | 0 | 32 | | 20 | | 2 | | 0 | 3 | 40 | | 7 | | 4 | | 0 |  |  |  |
| EGFR | - | | + | | ++ | | +++ | | - | | + | | ++ | | +++ | | Chi-square | 54 | 0.98 |
|  | 31 | | 17 | | 5 | | 1 | | 31 | | 18 | | 4 | | 1 | |  |  |  |
| Ki67 | Mean = 28.86 | | | | | | | | Mean = 21.52 | | | | | | | | Wilcoxon | 88 | <0.001 * |
| CK5/6 | - | | | | + | | | | - | | | | + | | | | Chi-square | 54 | 0.49 |
|  | 43 | | | | 11 | | | | 40 | | | | 14 | | | |  |  |  |
| AR | Mean = 32.95 | | | | | | | | Mean = 32.97 | | | | | | | | Wilcoxon | 64 | 0.81 |
| p53 | Mean = 22.42 | | | | | | | | Mean = 22.65 | | | | | | | | Wilcoxon | 55 | 0.94 |

n is the number of patients having paired information (both before and after NAC) therefore used for the according analysis.
