# Supplementary material for: Comprehensive evaluation of the relationship between biomarker profiles and neoadjuvant chemotherapy outcomes for breast cancer patients
Source: Diagn Pathol. 2024 Mar 20;19:53. doi: 10.1186/s13000-024-01451-y (PMC10953119; doi:10.1186/s13000-024-01451-y)
Supplement: Supplementary file 5 — Additional file 5: Supplementary Table 2. Comparison of biomarker profiles before and after NAC for HER2 positive patients. [file 13000_2024_1451_MOESM5_ESM.docx]

Supplementary Table 2: Comparison of biomarker profiles before and after NAC for HER2 positive patients

|  | Before NAC | | | | | | | | After NAC | | | | | | | | Test | n | p-value |
| --- | --- | --- | --- | --- | --- | --- | --- | --- | --- | --- | --- | --- | --- | --- | --- | --- | --- | --- | --- |
| ER | Mean = 34.62 | | | | | | | | Mean = 32.21 | | | | | | | | Wilcoxon | 58 | 0.14 |
| ER | - | | | | + | | | | - | | | | + | | | | Chi-square | 58 | 0.56 |
|  | 20 | | | | 38 | | | | 23 | | | | 35 | | | |  |  |  |
| PR | Mean = 18.91 | | | | | | | | Mean = 16.09 | | | | | | | | Wilcoxon | 58 | 0.027 * |
| PR | - | | | | + | | | | - | | | | + | | | | Chi-square | 58 | 0.58 |
|  | 25 | | | | 33 | | | | 28 | | | | 30 | | | |  |  |  |
| TOPO II | 0 | 1 | | 2 | | 3 | | 4 | 0 | 1 | | 2 | | 3 | | 4 | Chi-square | 30 | 0.75 |
|  | 1 | 11 | | 15 | | 3 | | 0 | 1 | 13 | | 11 | | 4 | | 1 |  |  |  |
| EGFR | - | | + | | ++ | | +++ | | - | | + | | ++ | | +++ | | Chi-square | 34 | 0.35 |
|  | 14 | | 14 | | 4 | | 2 | | 16 | | 11 | | 7 | | 0 | |  |  |  |
| Ki67 | Mean = 45.18 | | | | | | | | Mean = 35.91 | | | | | | | | Wilcoxon | 57 | 0.005 * |
| CK5/6 | - | | | | + | | | | - | | | | + | | | | Chi-square | 32 | 0.27 |
|  | 25 | | | | 7 | | | | 21 | | | | 11 | | | |  |  |  |
| AR | Mean = 33.94 | | | | | | | | Mean = 23.66 | | | | | | | | Wilcoxon | 35 | 0.010 * |
| p53 | Mean = 30.31 | | | | | | | | Mean = 30.88 | | | | | | | | Wilcoxon | 32 | 0.97 |

n is the number of patients having paired information (both before and after NAC) therefore used for the according analysis.
